# A rare invader on a cardiac stent: *Mycobacterium abscessus* mediastinitis managed with tedizolid after linezolid intolerance

**DOI:** 10.1016/j.idcr.2026.e02719

**Published:** 2026-08-04

**Authors:** Preeti S. Ajapuje, Shiv Gupta, Geeta Chitre, Samvveda Samel

**Affiliations:** a Department of Infectious disease, Inlaks and Budhrani Hospital, Pune. Maharashtra, India; b Department of Cardiac Surgery, Inlaks and Budhrani Hospital, Pune, Maharashtra, India; c Department of Microbiology, Inlaks and Budhrani Hospital, Pune, Maharashtra, India; d Department of Internal Medicine, Samvvudra Clinic, Mumbai, Maharashtra, India

**Keywords:** *Mycobacterium abscessus*, Tedizolid, Coronary stent infection, Mediastinitis, Percutaneous coronary intervention, Nontuberculous mycobacteria, Linezolid intolerance, Cardiac PET-CT

## Abstract

**Background:**

*Mycobacterium abscessus* infection involving coronary stent material and mediastinal tissues after percutaneous coronary intervention is exceptionally uncommon, difficult to diagnose, and challenging to treat because of biofilm formation, multidrug resistance, and the need for prolonged multidrug therapy.

**Case summary:**

A 65-year-old man with diabetes, hypertension, and coronary artery disease developed persistent fever after PCI to the left anterior descending and right coronary arteries. Serial cardiac PET-CT and contrast-enhanced CT demonstrated a hypermetabolic middle mediastinal mass encasing the left main coronary artery and proximal branches, with a pseudoaneurysm arising from the proximal LAD. He underwent removal of the infected coronary stent, coronary artery bypass grafting, pericardiectomy, and biopsy/debridement of the mediastinal lesion. Tissue culture grew *M. abscessus subsp. abscessus*, identified by MALDI-TOF, with susceptibility to amikacin and linezolid and resistance to macrolides and fluoroquinolones.

**Intervention and outcome:**

The patient received a multidrug regimen anchored by amikacin and an oxazolidinone. Serum creatinine and audiometry were monitored for IV amikacin. Because of early hematologic intolerance to linezolid, therapy was transitioned to tedizolid 200 mg once daily, with clofazimine later used as the companion oral agent after intolerance to minocycline. The patient completed a total of 6 months of anti-NTM therapy, including approximately 5 months of tedizolid-based therapy. The follow-up monitoring sheets document stable hematological parameters and no peripheral neuropathy observed during the tedizolid phase. Serial imaging showed substantial regression of the mediastinal lesion without clinically significant FDG uptake at end of therapy.

**Conclusion:**

This case highlights three clinically relevant lessons: first, post-PCI *M. abscesses* stent-associated mediastinitis demands aggressive source control; second, tedizolid can serve as a viable salvage therapy after linezolid intolerance; third, serial cardiac PET-CT can assist treatment monitoring when repeat deep tissue sampling is impractical; and fourth, advanced multimodal imaging plays a critical role in diagnosis, surgical planning, and monitoring of complex post-cardiac surgical infections.

**Key message:**

This case supports tedizolid as a pragmatic salvage oxazolidinone when prolonged therapy is required for deep-seated *M. abscessus* cardiovascular infection and linezolid-related hematologic toxicity threatens regimen completion, while also underscoring that cure depended on decisive surgical source control and meticulous longitudinal monitoring.

## Introduction

Coronary stent infection after percutaneous coronary intervention is rare but potentially catastrophic. Historical reviews suggest that late-onset coronary stent infection is associated with high mortality and usually requires combined medical and surgical management rather than antibiotics alone. In parallel, invasive cardiovascular infection due to rapidly growing mycobacteria is uncommon, diagnostically delayed, and notoriously difficult to eradicate because of biofilm formation on prosthetic material.

Nontuberculous mycobacteria (NTM) are environmental organisms increasingly recognized as causes of healthcare-associated infections. Among the rapidly growing mycobacteria (RGM), *Mycobacterium abscessus* is notoriously difficult to treat due to its intrinsic resistance to most standard antitubercular drugs and common antibiotics [1], [2]. While cutaneous and pulmonary infections are more frequently described [3], invasive cardiovascular infections following procedures such as percutaneous coronary intervention (PCI) are exceedingly rare and associated with high morbidity and mortality [4].

Current guidelines for treating *M. abscessus* generally recommend a multidrug regimen containing a macrolide, amikacin, and either cefoxitin or imipenem, often supplemented with oral agents such as oxazolidinones [1], [5]. Linezolid, the first-in-class oxazolidinone, is effective but its long-term use is frequently limited by significant adverse effects, including reversible myelosuppression (anemia, thrombocytopenia) and peripheral neuropathy, which typically occur after 2–4 weeks of therapy [6]. Tedizolid phosphate, a second-generation oxazolidinone, has demonstrated potent in vitro activity against *M. abscessus,* with minimum inhibitory concentrations (MICs) often 2- to 4-fold lower than linezolid [7]. Although clinical data on its long-term use in deep-seated NTM infections remains limited, emerging evidence suggests it may offer a more favorable safety profile [8], [9].

### Case presentation

A 65-year-old man with diabetes mellitus (most recent HbA1c 7.2%), hypertension, and coronary artery disease underwent PCI to the LAD and RCA. Approximately one week later he developed persistent low-grade fever.

Over the subsequent six months, empirical antibacterial and antitubercular therapy failed to produce durable improvement. Aside from the persistent low-grade fever, a comprehensive review of systems was notable for the absence of cough, dyspnea, chest pain, night sweats, weight loss, or any localizing neurological or gastrointestinal symptoms. The initial empirical antibacterial regimen included vancomycin and piperacillin-tazobactam, which was started empirically for possible healthcare-associated mediastinitis. Antitubercular therapy (rifampin, isoniazid, ethambutol, and pyrazinamide) was added because the patient resided in a region with intermediate tuberculosis endemicity, and the initial CT chest showed mediastinal lymphadenopathy with a central low-attenuation area that was radiographically suspicious for tuberculous mediastinitis. This differential diagnosis was further considered in the context of the patient's diabetes.

**Fig. 1:**
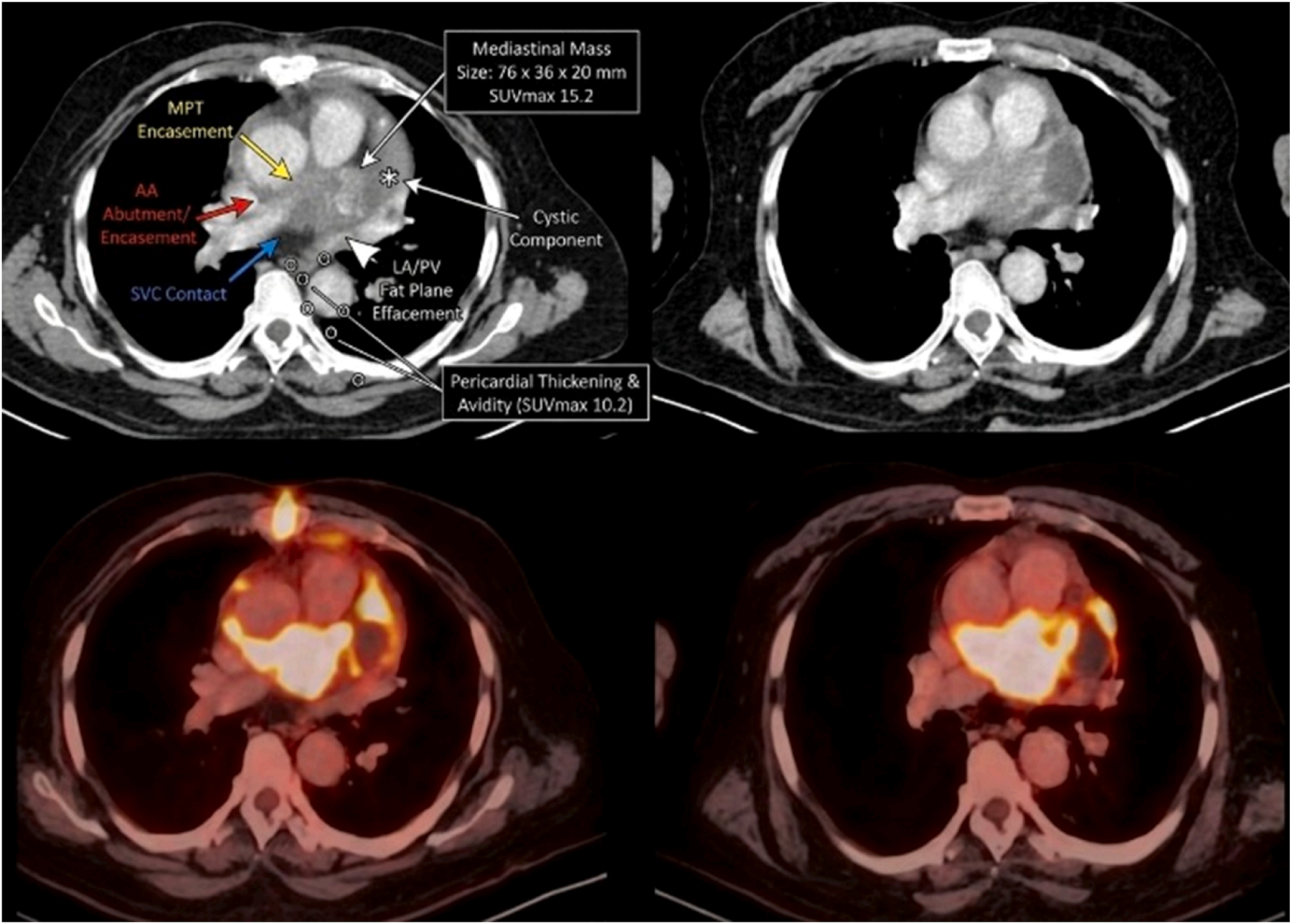
Baseline pre-operative serial cardiac PET-CT scan. (A) Correlation axial CT image at the level of the mediastinum showing the ill-defined heterogeneously enhancing mass. (B) Correlation fused PET-CT image demonstrating increased FDG uptake within the mass. (C) Axial CT image with anatomical annotations. (D) Annotated master fused PET-CT image. The mass (76 × 36 × 20 mm, SUVmax 15.2) partially encases the main pulmonary trunk (MPT) and ascending aorta (AA), contacts the superior vena cava (SVC) without luminal infiltration, abuts the left atrium and pulmonary veins (LA/PV) with effaced fat planes, and contains a hypodense cystic component along its left lateral margin. Diffuse heterogeneous increased FDG uptake is seen in the mildly thickened pericardium (SUVmax 10.2).

Cardiac PET-CT. performed approximately six months after symptom onset demonstrated a hypermetabolic middle mediastinal lesion A standard contrast-enhanced CT of the chest performed at symptom onset had already revealed mediastinal lymphadenopathy; the subsequent PET-CT, performed after failure of initial therapies, showed a heterogeneously enhancing middle mediastinal soft-tissue mass measuring 8.4 × 4.2 × 4.0 cm, centered to the left of the ascending aorta, with close relation to and reported encasement of the left main coronary artery and proximal LAD/circumflex branches. The lesion also abutted the proximal pulmonary arteries, partially compressed the lower superior vena cava, scalloped the anterosuperior wall of the left atrium, and displaced the left superior pulmonary vein posteriorly. A contrast-opacified 9 × 8 × 7 mm outpouching arising from the proximal LAD was reported as most consistent with pseudoaneurysm. Internal non-enhancing areas with rim enhancement were also described, together with a small adjacent pericardial effusion (Fig. 1).

**Fig. 2:**
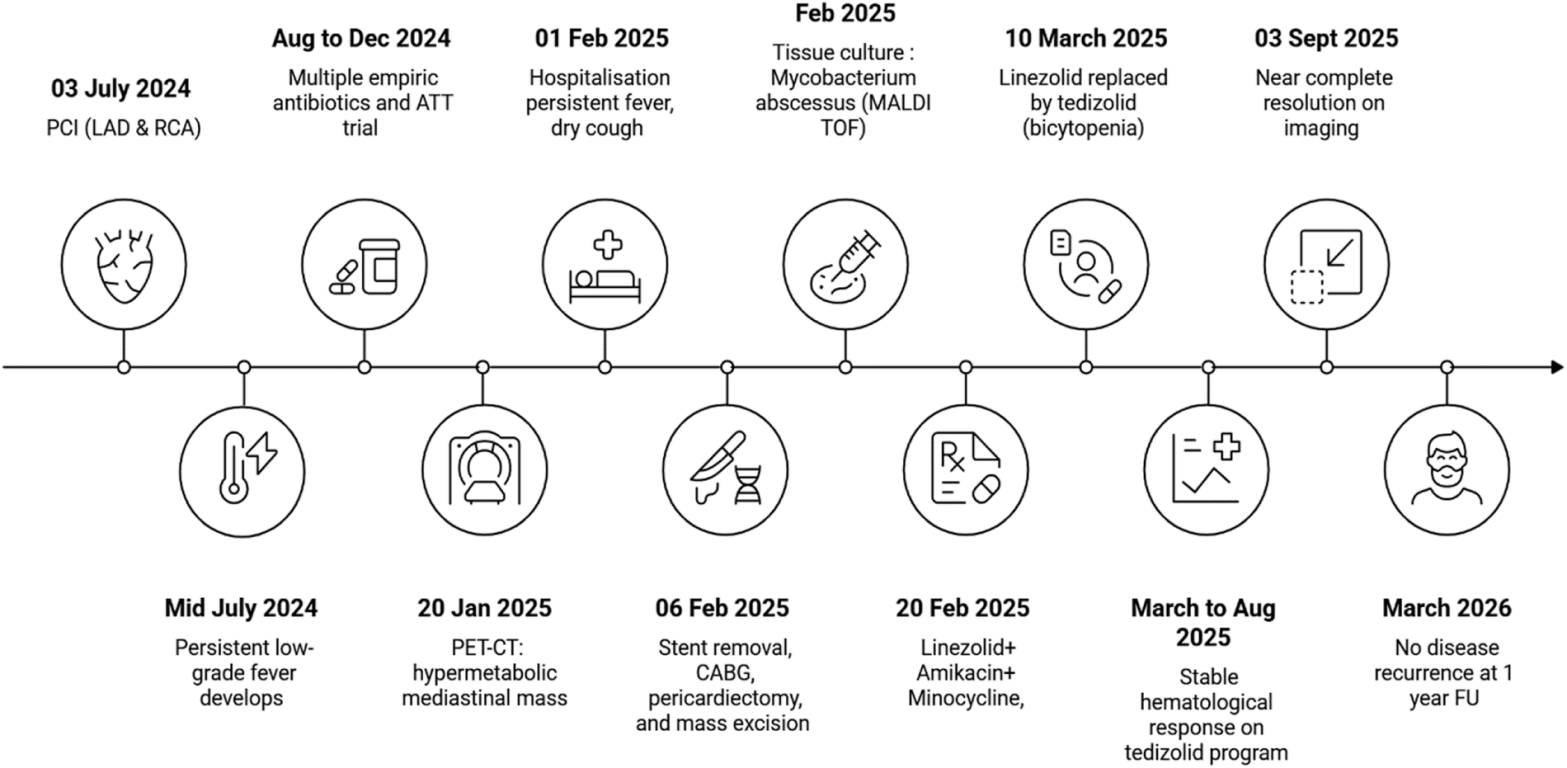
Clinical timeline of post-PCI *Mycobacterium abscessus* stent-associated mediastinal infection. Key clinical events from index procedure (03 July 2024) through surgical intervention (06 February 2025) and completion of antimicrobial therapy (September 2025) are illustrated chronologically. PCI, percutaneous coronary intervention; LAD, left anterior descending artery; RCA, right coronary artery; CABG, coronary artery bypass grafting; SVG, saphenous vein graft; PET-CT, positron emission tomography-computed tomography; FDG, fluorodeoxyglucose.

Because of persistent symptoms, the proximity to major cardiovascular structures, and concern for infected coronary hardware, the patient underwent removal of the coronary stent, CABG with saphenous vein graft (SVG) to LAD, pericardiectomy, and biopsy/debridement of the retro aortic or intrapericardial mass (Fig. 2). Histopathology showed caseous necrosis with ill formed granuloma with chronic inflammatory changes.

Tissue culture in MGIT medium subsequently grew *Mycobacterium abscessus* subsp. abscessus, with identification by MALDI-TOF. Broth microdilution susceptibility testing demonstrated activity of amikacin and linezolid, intermediate activity of cefoxitin and imipenem, and resistance to clarithromycin and fluoroquinolones (Table 1). Intraoperative tissue cultures were also performed for aerobic, anaerobic, and fungal organisms; all remained sterile after extended incubation, helping to rule out polymicrobial co-infection. Formal inducible macrolide resistance testing and erm(41) genotyping were not performed due to limitations of the reference laboratory at the time of testing, a limitation we acknowledge.

**Table 1:** Drug susceptibility profile of the *M. abscessus* isolate.

**Drug**	**MIC**	**Interpretation**
Amikacin	8 ug/mL	Susceptible
Linezolid	4 ug/mL	Susceptible
Cefoxitin	32 ug/mL	Intermediate
Imipenem	16 ug/mL	Intermediate
Clarithromycin (14 day incubation)	16 ug/mL	Resistant
Ciprofloxacin	4 ug/mL	Resistant
Moxifloxacin	8 ug/mL	Resistant
Doxycycline	16 ug/mL	Resistant
Minocycline	8 ug/mL	No CLSI breakpoint / IE
Tigecycline	2 ug/mL	No CLSI breakpoint / IE

Footnote: Specimen: aortic tissue from retro-aortic/intrapericardial mass. Species identification by MALDI-TOF mass spectrometry (Bruker Biotyper Sirius). Drug susceptibility testing (DST) by broth microdilution (Sensititre, Thermo Fisher Scientific). Clarithromycin incubated for 14 days per CLSI M24S guidelines to detect inducible macrolide resistance. Interpretation per CLSI M24S (February 2023). IE = Insufficient evidence for clinical breakpoints.

The patient's weight was 78 kg (BMI 26.4 kg/m²), and creatinine clearance was 89 mL/min. The initial antimicrobial regimen was constructed based on susceptibility results: intravenous amikacin (dosed at 15 mg/kg based on ideal body weight, adjusted for renal function), linezolid 600 mg orally twice daily, cefoxitin 2 g intravenously every 6 h, and clarithromycin 500 mg orally twice daily (despite in vitro resistance, as part of a combination strategy for potential synergy). This regimen was chosen in accordance with guideline recommendations for *M. abscessus* treatment, utilizing at least three active agents in the initial phase. Linezolid was discontinued after two weeks due to trilineage cytopenia (anemia, leukopenia, and thrombocytopenia). A trial of holding linezolid was attempted, followed by rechallenge at a reduced dose of 600 mg once daily; however, this again led to recurrent cytopenias. Therapeutic drug monitoring for linezolid was not available at our institution. Accordingly, therapy was switched to tedizolid 200 mg once daily. Serum creatinine and audiometry were monitored for IV amikacin. Minocycline was later replaced by clofazimine because of gastrointestinal intolerance. The oral tedizolid-based therapy was continued for 5 months with a total treatment duration of approximately 6 months of anti-NTM therapy.

**Fig. 3:**
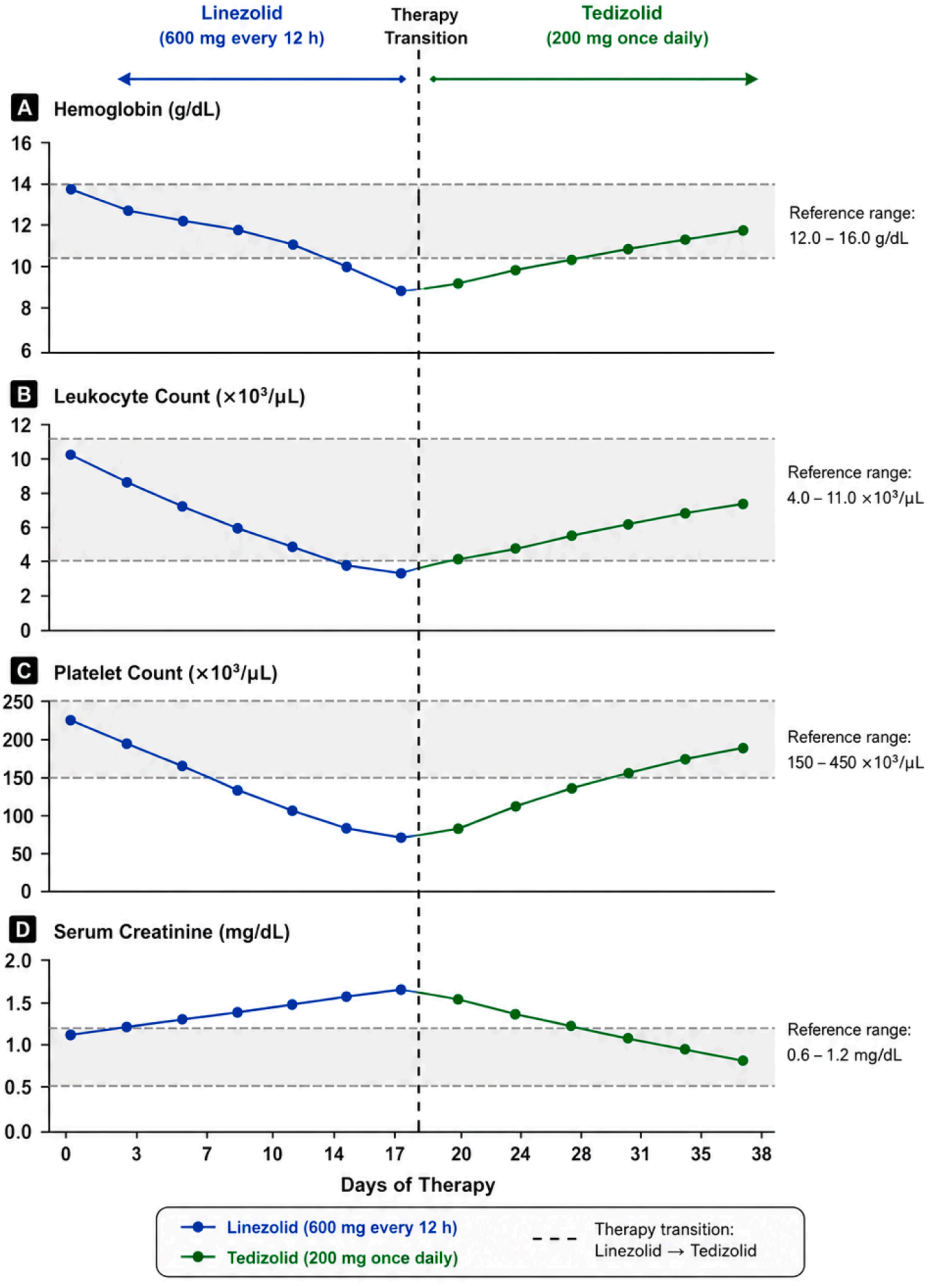
Serial laboratory monitoring during tedizolid-based therapy. Trends in laboratory parameters before and after transition from linezolid to tedizolid. The vertical dashed line indicates the switch from linezolid (600 mg every 12 h) to tedizolid (200 mg once daily) on Day 17. Shaded gray areas represent reference ranges for each parameter. (A) Hemoglobin (g/dL), (B) Leukocyte count (×10 ³/µL), (C) Platelet count (×10 ³/µL), (D) Serum creatinine (mg/dL). Following the transition, all parameters showed improvement toward or within their respective reference ranges.

The patient experienced easy bruising and fatigue during the period of linezolid-induced cytopenias, which resolved following the switch to tedizolid. The monitoring sheets showed stable hematological parameter and absence of peripheral neuropathy on follow-up as illustrated in Fig. 3.

### Outcome and follow-up

**Fig. 4:**
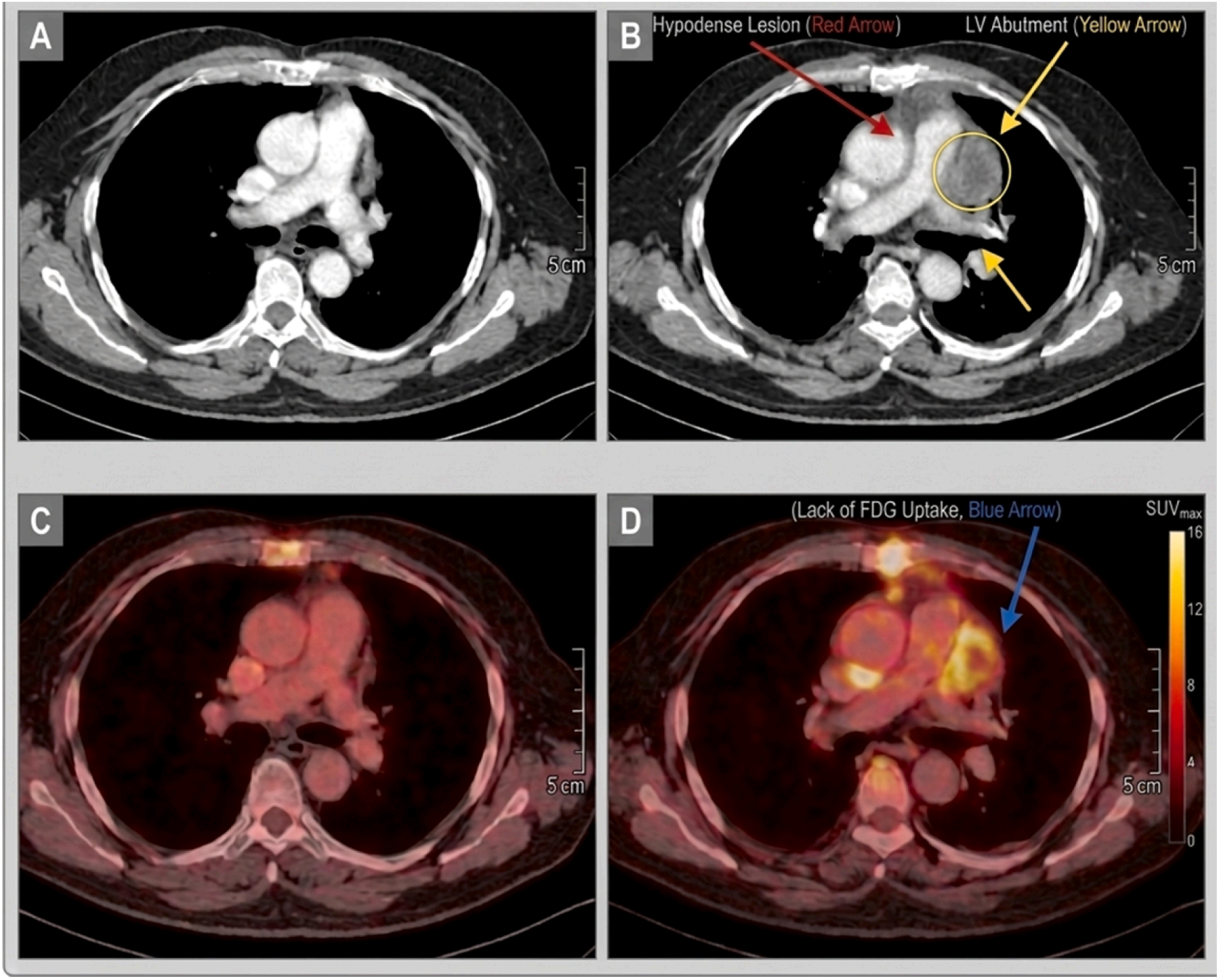
End-of-Therapy Serial Cardiac PET-CT. (A) Correlation axial CT image showing a small residual hypodense lesion (22 × 20 mm) in the middle mediastinum, left lateral aspect, abutting the left ventricle inferiorly (yellow arrow, red arrow). (B) Annotated correlation axial CT image with white circle highlighting the lesion. (C) Correlation fused PET-CT image demonstrating no significant FDG uptake within the lesion (blue arrow), comparable to adjacent mediastinal blood pool activity (sample SUVmax 2.1). (D) Annotated fused PET-CT image confirming lack of metabolically active disease. Findings are consistent with a complete metabolic response.

Serial imaging supported clinical response. A follow-up study approximately 5 weeks after surgery showed persistent but slightly smaller disease burden, whereas the end-of-therapy Cardiac PET-CT on 03 September 2025 documented near-complete resolution of the mass, with a small residual hypodense lesion measuring approximately 22 × 20 mm showing no clinically significant FDG uptake, consistent with metabolic remission (Fig. 4). The sternal wound healed, fever resolved, and the patient returned to normal daily activities.

At one year follow up, the patient remains asymptomatic with no clinical or radiological evidence of disease recurrence.

## Discussion

This case substantially augments the literature on tedizolid-containing therapy for nontuberculous mycobacterial disease and is particularly noteworthy for documenting successful treatment of a coronary stent-associated infection mediastinitis following PCI. The clinical message is less that tedizolid alone is curative and more that tedizolid enabled completion of a prolonged oxazolidinone-containing regimen after linezolid intolerance, in a setting where source control had already been achieved surgically.

The first management principle is source control. Published experience with coronary stent infection suggests that late presentations generally require surgery, and reports of *M. abscessus* endovascular stent infection likewise emphasize that removal of infected foreign material is essential for cure. The patient's clinical course and key management milestones are summarized in Fig. 2. Invasive *M. abscessus* infection after cardiac surgery is associated with prolonged therapy, frequent adverse drug events, repeated debridement, and poor mortality, underscoring the seriousness of this disease category.

The second management principle is careful antimicrobial construction. Guideline documents emphasize at least three active drugs in the initial phase of *M. abscessus* treatment, chosen with expert input and susceptibility guidance. In the present case, macrolides and fluoroquinolones were not viable because the isolate was resistant, leaving amikacin and an oxazolidinone as key active agents while companion oral drugs were selected pragmatically based on tolerance.

Why is tedizolid reasonable here? In vitro work consistently shows lower MIC distributions for tedizolid than for linezolid against *M. abscessus* complex, but that microbiologic signal must be interpreted cautiously because validated tedizolid breakpoints for mycobacteria are lacking. Zhang et al. demonstrated that tedizolid had greater in vitro potency than linezolid against clinical isolates of *M. abscessus* complex, with MIC50 and MIC90 values of 2 and 4 μg/mL, respectively, and the MICs of tedizolid and linezolid were positively correlated [11]. This microbiologic advantage provides a strong rationale for considering tedizolid when linezolid cannot be continued. Selected published experience with tedizolid-containing regimens in NTM disease is summarized in Table 2.A published case of *M. abscessus* CAPD-associated peritonitis and a case of prolonged cutaneous NTM infection both support tedizolid as a salvage option after linezolid-induced thrombocytopenia [9], [10], Importantly, in contrast to the report by Shaw et al. (where peripheral neuropathy was noted despite hematologic tolerance) and other series, our case demonstrated no hematologic toxicity (including no recurrence of bicytopenia) and no peripheral neuropathy during the entire 5 months of tedizolid therapy. This favorable tolerability profile, consistent with the known pharmacologic properties of tedizolid, further supports its role as a rational salvage therapy. We also acknowledge, however, that validated CLSI breakpoints for tedizolid against nontuberculous mycobacteria are not yet available, precluding formal interpretation of tedizolid MIC testing had it been performed.

**Table 2:** Selected published experience with tedizolid-containing regimens in nontuberculous mycobacterial disease.

**Reference**	**Clinical setting**	**Why tedizolid was used**	**Reported outcome**	**How it informs current case**
Zhang et al., [11]	*In vitro* study of 133 clinical NTM isolates	Not applicable (preclinical study)	TZD MIC50/90 for *M. abscessus:* 2/4 μg/mL; TZD more potent than LZD; MICs positively correlated	Provides strong *in vitro* rationale: lower MICs and positive correlation support TZD as a rational substitute when LZD cannot be continued
Yuste et al., [8]	Pulmonary NTM infection	Switch from linezolid because of hematologic toxicity and GI intolerance	Prolonged tedizolid use described after linezolid toxicity	Earliest published prolonged tedizolid experience in NTM; supports switch for hematologic intolerance.
Miyashita et al., [10]	*M. abscessus* CAPD-associated peritonitis	Linezolid-associated thrombocytopenia	Maintained on oral tedizolid; no recurrence reported at 1 year	Supports tedizolid-containing salvage therapy in *M. abscessus* infection, demonstrates favorable hematologic tolerance
Shaw et al., [9]	Cutaneous *M. chelonae* infection	Severe thrombocytopenia after 30 days of linezolid	8 months of tedizolid with clinical remission, but possible peripheral neuropathy/late relapse	In contrast, our case showed no peripheral neuropathy or hematologic abnormalities after 5 months of tedizolid

The third principle is response assessment. For deep-seated mediastinitis adjacent to major vessels and coronary structures, repeat culture is invasive and often impractical. In a previously published *M. abscessus* endovascular stent infection, cardiac PET-CT was specifically reported as useful when conventional imaging was insufficient. In the current case, serial cardiac PET-CT offered a clinically persuasive measure of response by distinguishing residual tissue from ongoing metabolically active infection.

This case has several strengths:

• Complete microbiologic characterization with species-level identification and CLSI-guided susceptibility testing
• Aggressive surgical source control with hardware removal
• Serial cardiac PET-CT monitoring of treatment response
• Successful salvage switch to tedizolid enabling prolonged therapy without recurrent toxicity
• Adherence to CARE reporting guidelines and one-year follow-up confirming sustained remission
• Robust evidence of durable treatment response
• The exceptional rarity of post-PCI *M. abscessus* mediastinitis means that individual case reports remain essential for building the evidence base to inform future clinical practice.

Two limitations also warrant acknowledgment. First, the concurrent administration of surgery and combination therapy precludes attribution of cure to any single intervention. Acknowledging the absence of formal erm(41) genotyping and inducible macrolide resistance testing is a limitation, as these data could have further contextualized the clarithromycin resistance pattern observed. Second, validated CLSI breakpoints for tedizolid against nontuberculous mycobacteria are not yet available, preventing formal interpretation of tedizolid MIC testing had it been performed.

## Conclusion

Post-PCI *M. abscessus* stent-associated mediastinitis is rare and carries a high morbidity and mortality burden. In this case, cure was most plausibly achieved through the combination of aggressive surgical source control, amikacin-based multidrug therapy, and successful completion of a prolonged tedizolid-containing oral phase after linezolid intolerance. Furthermore, this case underscores the critical role of advanced multimodal imaging, including cardiac PET-CT, in the diagnosis, surgical planning, and monitoring of complex post-cardiac surgical infections. Tedizolid should be presented as a rational salvage oxazolidinone supported by growing clinical experience.

## Abbreviations

AFB: Acid-fast bacilli

ATT: Antitubercular therapy

CABG: Coronary artery bypass grafting

CAD: Coronary artery disease

CECT: Contrast-enhanced computed tomography

CLSI: Clinical and Laboratory Standards Institute

DST: Drug susceptibility testing

FDG: Fluorodeoxyglucose

LAD: Left anterior descending artery

LZD: Linezolid

MALDI-TOF: Matrix-assisted laser desorption/ionization-time of flight

MGIT: Mycobacteria growth indicator tube

MIC: Minimum inhibitory concentration

NTM: Nontuberculous mycobacteria

PCI: Percutaneous coronary intervention

PET-CT: Positron emission tomography-computed tomography

RCA: Right coronary artery

RGM: Rapidly growing mycobacteria

SVG: Saphenous vein graft

SUVmax: Maximum standardized uptake value

TZD: Tedizolid

## Ethics

No formal ethics committee approval required for a single retrospective case report.

## Clinical transparency statement

The clinical management described was based entirely on patient need, published guidelines, and the treating physicians' clinical judgment, without any external influence or promotional intent regarding tedizolid use.

## Patient consent

Written informed consent for publication of the case details and images was obtained.

## Declaration of Competing Interest

The authors declare that they have **no known competing financial interests or personal relationships** that could have appeared to influence the work reported in this paper.

Specifically:

• **No financial support or funding** was received for this study, case report, or manuscript preparation.
• **No pharmaceutical company** provided tedizolid, linezolid, or any other drug free of charge for this patient's treatment.
• **No author has served** as a consultant, speaker, or advisory board member for any company manufacturing oxazolidinones (including Merck/Cubist, Pfizer, or any generic manufacturers of linezolid or tedizolid).
• **No author holds** stock, patents, or other equity interests in any relevant pharmaceutical or diagnostic company.
• **No author has received** travel grants, writing assistance, or honoraria related to this work.
• **No institutional or industry-sponsored grants** supported the microbiological testing, imaging, or surgical procedures described in this case.

## Data Availability

De-identified source data supporting the figures and tables can be made available on reasonable request, subject to institutional policy and patient confidentiality constraints.
